# Glacier Surface Motion Estimation from SAR Intensity Images Based on Subpixel Gradient Correlation

**DOI:** 10.3390/s20164396

**Published:** 2020-08-06

**Authors:** Li Fang, Zhen Ye, Shu Su, Jian Kang, Xiaohua Tong

**Affiliations:** 1 The Quanzhou Institute of Equipment Manufacturing, Haixi Institute, Chinese Academy of Sciences, Quanzhou 362216, China; fangli@fjirsm.ac.cn; 2 College of Surveying and Geo-informatics, Tongji University, Shanghai 200092, China; sushu@tongji.edu.cn (S.S.); xhtong@tongji.edu.cn (X.T.); 3 Research Institute of Electronic Engineering Technology, Harbin Institute of Technology, Harbin 150001, China; 13s105045@hit.edu.cn

**Keywords:** glacier surface motion, SAR intensity image, image matching, gradient correlation

## Abstract

With the current extensive availability of synthetic-aperture radar (SAR) datasets with high temporal (e.g., a repeat cycle of a few or a dozen days) and spatial resolution (e.g., in the order of ∼1 m), radar remote sensing possesses an increasing potential for the monitoring of glacier surface motion thanks to the nearly weather and time-independent advantages. This paper proposes a robust subpixel frequency-based image correlation method for dense matching and integrates the improved matching into a workflow of glacier surface motion estimation using SAR intensity images with specific pre-processing and post-processing steps. The proposed matching method combines complex edge maps and local upsampling in the frequency domain for subpixel intensity tracking, which ensure the accuracy and robustness of glacier surface motion estimation. Experiments were carried out with TerraSAR-X and Sentinel-1 images covering two glacier areas in pole and alpine regions. The results of the monitoring and investigation of glacier motion validate the feasibility and reliability of the presented motion estimation method based on subpixel gradient correlation. The comparative results using both simulated and real SAR data indicate that the proposed matching method outperforms commonly used correlation-based matching methods in terms of matching accuracy and the ability to obtain correct matches.

## 1. Introduction

As an important part of the albedo feedback mechanism of the local and global climate systems, glaciers cover approximately 10% of the Earth’s surface [1]. However, in the context of global warming, the melting of glaciers is predicted to make a greater contribution to global sea-level rise [2]. It is estimated that the melting of glaciers accounts for about 30% of the observed sea-level rise [3]. Therefore, in the past decade, studies on the behavior of glaciers and the mass balance of glaciers with geometric changes have attracted more and more attention. Since glaciers are continuous dense ice bodies that move under their own gravity, monitoring the glacier surface motion and its velocity is the basic method for obtaining quantitative changes in glaciers and provides digital clues to infer past glacier changes and predict future development [4].

In the early stage, the glacier studies depended on compulsory field surveys by glaciologists. To acquire sufficient and reliable measuring data, researchers are obliged to travel to distant glaciers, which are usually located in mountainous areas with highly hazardous environments. However, the collection of measuring field data can be conducted at dozens of positions at specific time points, which greatly depends on the weather condition and the situation of accessibility and takes much time and money. Therefore, obtaining reliable measurement data on a large-scale glacier has long been a difficult task [3]. To address this problem, remote sensing technology started to be an eligible and cost-effective solution in the past decades [5]. Remote sensing data, especially those acquired by space-borne platforms, provide high-resolution, reliable, large-scale, and chronological measurements in an efficient manner, which have proven to be the most adequate data sources for monitoring glaciers. There is a wide range of remote sensing data of different characteristics, sensors, and imaging modes. Among various remote sensing data, synthetic-aperture radar (SAR) data have the ability to ignore weather conditions and can provide images with intensity and phase information throughout the year, which is attracting more and more attention in measuring glacier motion [6].

With the launch of more advanced SAR satellites, such as TerraSAR-X (TSX) and Sentinel-1, the increasing availability of spaceborne SAR datasets can reach a short temporal baseline of a few or a dozen days and a high spatial resolution in the order of ∼1 m. The high temporal and spatial resolution of SAR data enables the fine-grained and detailed observation of glacier surface motion, and thus offers more potential possibilities and opportunities for glacier displacement monitoring with offset tracking. On the other hand, the high temporal and spatial resolution puts forward higher demand on the accuracy and robustness of offset tracking using SAR intensity images. Subpixel matching method is a decisive component of offset tracking, which largely dominates the final performance of motion estimation. In this study, we develop a robust subpixel frequency-based image correlation method, called locally upsampled gradient correlation (LUGC), to achieve reliable offset tracking of SAR intensity images, and validate the effectiveness in the applications of glacier surface motion estimation. Our contributions that are specific to monitoring the glacier surface motion include:

- An improved robust frequency-based image correlation method, which combines complex edge maps and local upsampling in the frequency domain for subpixel translation estimation, is introduced and integrated into a workflow of glacier surface motion estimation using SAR intensity images.
- The reliability and feasibility of the presented dense motion estimation method based on subpixel gradient correlation is demonstrated by using TSX and Sentinel-1A (S1A) images covering two glacier areas in pole and alpine regions.

## 2. Background of SAR Image-Based Glacier Motion Estimation

The optical images acquired by Earth observation satellites are the most commonly used datasets for glacier monitoring. However, the frequent cloud cover and sunlight variation in polar and high mountain areas inevitably make the permanent collection of optical data impossible, which leads to insufficient data coverage [7]. Contrary to optical observation, SAR imaging is a supplementary information source, which provides images throughout the year without being limited by weather conditions and imaging time. The current spaceborne SAR images (e.g., TSX Spotlight Mode) can resolve a ground sampling distance of up to nearly 1 m, which is eligible for the task of glacier monitoring [8,9]. Generally, the main methods of using SAR image data to monitor glacier dynamics can be divided into two categories: interferometric methods and offset tracking-based methods.

### 2.1. Interferometric Methods

Interferometric methods are regarded as an accurate technique for glacier monitoring, with the highest sensitivity to surface motion. With the aid of the subsequent phase changes or the sequence of transmitted wavelengths, it is possible to accurately measure the movement of surface feature patterns [10,11]. Differential interferometric SAR (D-InSAR) is a representative of InSAR methods, aiming to detect ground deformation from the difference between two or more repeated SAR image pairs [12]. D-InSAR technology has greatly promoted the development of glaciology by providing nearly the most precise accuracy [13]. It has been used in applications such as the interference of the Arctic and Antarctic velocity measurements of ice sheets and streams [14] and alpine glaciers [15]. However, when applying the D-InSAR method, it is worth noting that terrain information should be deleted from SAR data through an external digital elevation model (DEM) or multiple SAR pairs [3]. Multi-aperture InSAR (MAI) is an alternative InSAR technique that can overcome the one-way limitation of observation and infer azimuth displacement from the interference pair [6,16]. In [17], the authors compared the ice speed and glacier thickness of traditional InSAR and MAI. Apart from D-InSAR and MAI, coherent tracking is the last major category of techniques related to SAR interferometry. Interference coherence stands for the degree of phase correlation between a pair of single-look complex (SLC) products. The underlying assumption of this method is that losing consistency over moving glaciers is mostly the result of local co-registration errors. By adjusting the relative offset of these low-coherence regions until the local coherence is maximized, it can be speculated that the corresponding surface motion will also be captured more accurately [3]. To this end, multiple image sub-windows distributed on the SLC scene can be used to create a series of interferograms, and the coherence can be estimated accordingly. Coherent tracking is usually used in combination with other methods, such as D-InSAR and feature tracking [18].

### 2.2. Offset Tracking-Based Methods

Offset tracking-based methods usually use the amplitude information of SAR data, which is mainly carried out by image correlation for measuring offsets of points or features on glacier surfaces. Here, feature tracking and intensity tracking are two commonly used methods for offset estimation.

Feature tracking relies on the detection of continuous, co-registered image centering on the movement of identifiable objects (e.g., cracks, grooves) or feature points on the surface of a glacier, which can derive a two-dimensional velocity field. Feature tracking methods can be achieved by either manual selection or automatic tracking. In the early days, feature tracking was mainly performed manually [19], and currently, the selection of features and the following tracking are done automatically by feature operators [20,21]. The premise of these approaches is that during the observation time, these features would remain fairly undisturbed on the glacier. The relative displacements measured between the respective features can then provide estimates of motion during the observation time [3]. Point-like features or the scattering characteristics of coherent scatterers during imaging are very stable and hardly change over time, which can be used for tracking [22]. In [23], the authors proposed a new coherent scattering detection algorithm, called the generalized likelihood ratio test method, which can show better performance when dealing with natural scenarios. Speckle tracking can be seen as enhancing the detection of SAR features. Unlike standard tracking of SAR features, this approach does not include recognizable objects or visible feature points on the glacier’s surface, but uses the SAR image speckle pattern. It is a high-frequency noise-like phenomenon that occurs in all coherent measurement systems and can be related to pairs of SAR images under some conditions. Usually, the tracking of the spot pattern is mainly achieved by a matching algorithm based on correlation [24]. Still, in some cases, the spot tracking can also use the amplitude and phase information of the SAR data [3]. To ensure robustness, in [25], the authors also addressed the pros and cons of using the SAR data for sophisticated local knowledge. Additionally, the benefits of tracking both standard features and speckle patterns are combined into one method to achieve the promising monitoring results [26].

Intensity tracking is commonly based on image correlation methods that calculate the similarity between small sliding templates to obtain the relative displacements between image pairs in the distance and azimuth directions, respectively. This technology was first introduced into the field of optical data [27] and was later widely used in applications based on SAR images [18]. Among the related algorithms, normalized cross-correlation (NCC) [28] and its variants calculated in the frequency domain [29,30] are the most popular methods to find the corresponding template. However, if there is no distinct pattern or texture on the surface of the glacier to obtain reliable correlation, the matching result may be greatly damaged. Phase correlation (PC) is a Fourier-based matching technique, which is considered to be more accurate and more effective than popular correlation-based methods such as the aforementioned NCC [31,32]. Several researchers have implemented PC-based methods to match SAR images across a broad variety of applications. In [33], a comparison of intensity tracking and interferometry for monitoring the flow of glaciers was given, in which the PC performs better on a fast ice flow, and the time variation of glacial scattering is more stable and uses only one pair of images. In some cases, PC-based Fourier–Merlin transformation is applied to single-frequency, repeated-pass, and dual-frequency SAR images for precise registration [34]. In [35], the traditional PC method was used to detect and calculate sea ice motion to achieve pixel-level accuracy measurement. However, the PC-based method used for SAR images still encounters some problems. For example, the work of Komarov and Barber [36] achieved sea ice motion with integer pixel precision through PC matching, which is lower than the standard NCC algorithm with sub-pixel accuracy. In [8], point-like features were combined with subpixel PC for an accurate estimation of the glacier surface motion. Nevertheless, the intensities of SAR images are often polluted by multiplicative noise, such as speckles in the high-frequency domain, which is not conducive to PC findings being robust. Furthermore, the large and discontinuous deformation of the glacier surface also hinders the calculation using the correlation-based matching methods. Therefore, the image correlation method involved must be both robust and accurate in order to be suitable for glacier monitoring along with SAR images.

## 3. Methodology

### 3.1. Workflow of Glacier Surface Motion Estimation Using SAR Intensity Images

**Figure 1:**
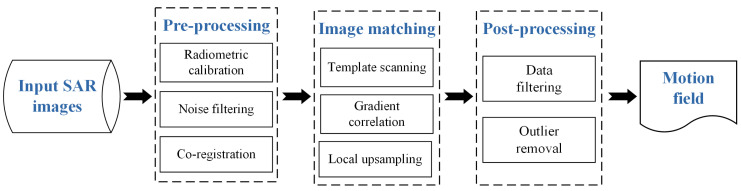
Workflow of glacier surface motion estimation using SAR intensity images.

Similar to the conventional scheme of Earth surface dynamics monitoring from optical images [4,37,38], the adopted workflow of glacier surface motion estimation using SAR intensity images includes three basic steps including pre-processing, dense matching, and post-processing, which are shown in Figure 1.

The purpose of the pre-processing step is to enhance the image radiometric quality, as well as to co-register the input images acquired from different times. With the inputs of SAR intensity images, the radiometric task is performed through radiometric calibration implemented using ENVI/SARscape software (http://www.sarmap.ch/wp/index.php/software/sarscape/) following the radar equation principle, speckle noise filtering with an adaptive Lee filter [8], and multi-look processing if necessary, whereas the co-registration task is performed by geocoding and orthorectification using available DEM.

The dense matching step, also called intensity tracking, uses a sliding template scanning through the dense grid defined on the master image with a certain step and finds the best offsets between the corresponding templates by maximizing a similarity measure. The output is two 2D subpixel-level displacement maps in both the *x*- (east-west) and *y*- (north-south) directions. In this study, we calculate the similarity between complex gradient representations and adopt frequency-based image correlation by means of Fourier transform (FT) [32] to accelerate the intensity tracking.

There inevitably exist errors and outliers in the results of dense matching due to the non-perfect image and scene conditions, and the stationary areas other than the glacier are not the focus of ice velocity monitoring. Therefore, the post-processing step includes data filtering and outlier removal [39]. Firstly, the results outside glacierized areas are removed using a mask generated manually or from digital glacier outlines, and the abnormal displacement vectors beyond assumed maximum values are eliminated. The remaining erroneous measurements are further filtered out depending on the neighborhood. Only the displacements that deviate less than a certain threshold from the mean filtering of a 3×3 neighborhood in both directions are retained [40,41]. Finally, the isolated points with less than 40% of valid points in their direct vicinity are removed.

### 3.2. Dense Matching Based on Subpixel Gradient Correlation

**Figure 2:**
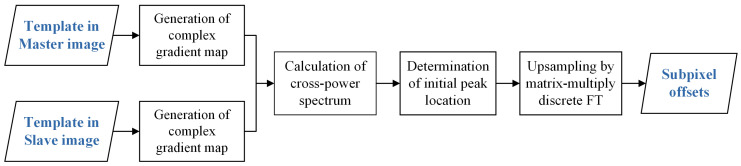
Flowchart of the locally upsampled gradient correlation (LUGC) method.

Dense matching is the core part of the workflow, the performance of which directly dominates the final quality of motion estimation. In order to ensure the matching accuracy and reliability in the presence of the complicated noises of SAR intensity images, a robust subpixel method called LUGC is developed in this study. The cross-correlation is calculated with the intensity gradients and gradually upsampled to a desired resolution, both in the frequency domain. For matching templates on each grid position, the flowchart of the LUGC method for subpixel offset estimation is illustrated in Figure 2, and the details are described in the following.

#### 3.2.1. Gradient Correlation

Gradient correlation replaces the original image intensities with gradient representations in the process of frequency-based cross-correlation [42]. The gradient representations of input templates $G_{f}$ and $G_{g}$ are constructed in the form of complex terms, whose real and imaginary parts are calculated as the bidirectional image gradients:

(1) $$\begin{array}{cc} \; & G_{f}\left(x,y\right)=G_{f,x}\left(x,y\right)+iG_{f,y}\left(x,y\right)\\ \; & G_{g}\left(x,y\right)=G_{g,x}\left(x,y\right)+iG_{g,y}\left(x,y\right) \end{array}$$

where $G_{f/g,x}$ and $G_{f/g,y}$ denote the gradients along the horizontal and vertical directions, respectively, and *i* is the complex imaginary unit. The intensity gradients can be approximated using first-order or second-order central differences as $G_{f/g,x}=h_{x}⋆I_{f/g}$ and $G_{f/g,y}=h_{y}⋆I_{f/g}$, where $h_{x}$ and $h_{y}$ are the approximated filters.

According to the correlation theorem, the cross-correlation can be operated in the frequency domain as the product of the FT of one image and the complex conjugate of the FT of the other [43]. Therefore, the correlation function of gradient correlation, defined as the inverse FT of the cross-power spectrum between the gradient representations, is given by:

(2) $$C_{GC}=F^{-1}\left\{F_{G_{f}}\left(u,v\right)F_{G_{g}}{\left(u,v\right)}^{*}\right\}$$

where $F_{G_{f}}\left(u,v\right)$ and $F_{G_{g}}\left(u,v\right)$ are the corresponding FT of $G_{f}\left(x,y\right)$ and $G_{g}\left(x,y\right)$, $F^{-1}$ denotes the inverse FT operator, and ∗ denotes the complex conjugate operator. Assume that two templates in master and slave images are related by offsets Δx and Δy in two directions as g(x,y)=f(x−Δx,y−Δy). According to the translation property of FT:

(3) $$F_{g}\left(u,v\right)=F_{f}\left(u,v\right)exp\left\{-i(u\Delta x+v\Delta y)\right\}$$

Then, gradient correlation in the frequency domain can be expressed as:

(4) $$\begin{array}{cc} \; & \hat{GC}=F_{G_{f}}\left(u,v\right)F_{G_{g}}{\left(u,v\right)}^{*}=M_{G}\left(u,v\right)exp\left\{-i(u\Delta x+v\Delta y)\right\}\\ \; & M_{G}\left(u,v\right)={\left|\left\{F_{h_{x}}\left(u,v\right)+iF_{h_{y}}\left(u,v\right)\right\}F_{f}\left(u,v\right)\right|}^{2} \end{array}$$

where the last term $exp\left\{-i(u\Delta x+v\Delta y)\right\}$ is the phase difference term, which contains the offset information and is the theoretical basis of PC [44]. Therefore, compared with standard cross-correlation and PC, gradient correlation in the frequency domain weights the phase difference term by a bandpass filtered term, considering the bandpass spectral selection property of central differences to estimate gradients [45]. Compared with orientation correlation (OC) [5,29], gradient correlation combines the magnitude and orientation of image gradients. The bandpass property and the joint use of magnitude and orientation information not only emphasize the frequency response of salient features, but also reduce the influence of high-frequency noise and aliasing, which can facilitate image correlation in the case of complicated scenarios [46].

#### 3.2.2. Local Upsampling in the Frequency Domain

Similar to the standard cross-correlation, the pixel-level offset results of gradient correlation can be determined by finding the peak location of the correlation function defined in Equation (2). However, subpixel measurements are normally pursed in order to improve the performance of surface displacement and velocity estimation. In [47], three subpixel approaches, namely intensity interpolation, similarity interpolation, and similarity fitting, were evaluated in surface displacement measurements, and intensity interpolation and similarity interpolation have been found to perform better. However, these approaches are considerably computationally expensive especially for large scaling factors and large covers. For the frequency-based image correlation methods, we can alternatively upsample the computed cross-power spectrum to a higher resolution in the frequency domain, since the correlation of two upsampled images is equivalent to upsampling the correlation of two original images [48]. There are two manners to achieve frequency-based upsampled cross-correlation: zero-padding and matrix-multiply discrete FT [49,50]. The former is realized by extending the cross-power spectrum and inserting zero frequencies in the middle, which is equivalent to interpolating the corresponding time signal, and the latter is realized by means of discrete FT implementation in the form of matrix multiplication.

Increasing the upsampling factor can enhance the subpixel precision of this approach, but largely increase the memory and computational burden if directly interpolating the global cross-power spectrum. Therefore, a coarse-to-fine local upsampling strategy that is able to handle a large upsampling factor is adopted for subpixel matching with gradient correlation [51,52].

In the coarse step, an initial estimate is obtained by finding the peak location of the gradient correlation function globally upsampled using zero-padding with an initial upsampling factor $k_{0}$.

The following step solely oversamples the cross-power spectrum of gradient correlation in a very small neighborhood around the initial peak location and avoids zero-padding the entire range. In this step, matrix multiplication implementation is more suitable for local upsampling. The gradient correlation function locally upsampled in a neighborhood of s×s pixels with a upsampling factor *k* can be calculated by multiplying three matrices:

(5) $$\begin{array}{cc} C_{LUGC}\left[X^{\prime },Y^{\prime }\right] & =exp\left(\frac{i2\pi Y^{\prime }V^{T}}{Hk}\right)\cdot \hat{GC}\left[U,V\right]\cdot exp\left(\frac{i2\pi UX^{{}^{\prime }T}}{Wk}\right)\\ \; & =Mat1_{sk\times H}\cdot Ma2_{H\times W}Mat3_{W\times sk} \end{array}$$

where $\hat{GC}\left[U,V\right]$ is the original cross-power spectrum matrix of gradient correlation, *W* and *H* are the template width and height, and $\left[X^{\prime },Y^{\prime }\right]$ and $\left[U,V\right]$ are the spatial distributions in the upsampled grid and the frequency distributions in the original grid, respectively. With this upsampled gradient correlation function, the final fractional offsets can be determined by relocating the peak at the new resolution with a subpixel precision of 1/*k*. In this study, we set the initial upsampling factor $k_{0}$, the final upsampling factor *k*, and neighborhood size *s* as 2, 50, and 1.5.

## 4. Experiments

### 4.1. Study Area and Dataset

In this study, two glaciers in alpine and pole regions were selected as the study areas to assess the proposed method and to measure the real glacier motion. The detailed information of the used image data can be seen in Table 1.

**Figure 3:**
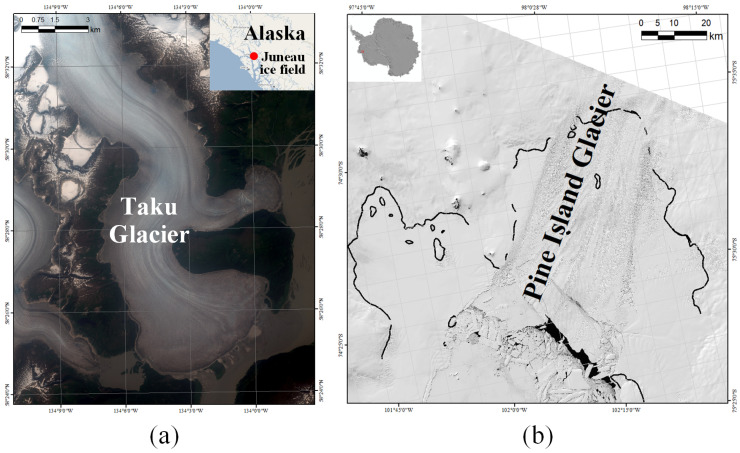
Illustration of the study area. (**a**) Taku Glacier; and (**b**) Pine Island Glacier.

- Taku Glacier is the principal outlet glacier of the Juneau Icefield located in the Coast Mountains of southeast Alaska, as shown in Figure 3a. It is one of the deepest and thickest temperate glaciers known in the world with the maximum thickness measured at 1477 m and the length of the ice shelf approximately reaching 58 km [53]. Taku Glacier is an advancing tidewater glacier characterized by high accumulation rates and very high melt rates at low elevations with a large mass turnover [54]. The behavior of Taku Glacier can greatly reflect the tendency of changes in the Juneau Icefield, possessing significant research value.
  A pair of TSX images covering the lower Taku Glacier was employed. The image pairs were acquired in strip-map mode from ascending pass direction and have an HH polarization. The imaging dates were 2009.07.11 and 2009.07.22 with one repeat cycle of 11 days apart. The spatial resolution was resampled to 2.09 m × 2.09 m in geometric pre-processing.
- Pine Island Glacier is one of the largest and fastest glaciers in West Antarctica, as shown in Figure 3b. As one of the major contributors to sea level rise, Pine Island Glacier has gained tremendous attention [55,56]. It has been undergoing thinning and retreat. The ice velocity of Pine Island Glacier accelerated from ∼2.8 km a${}^{-1}$ in 1996 to ∼4 km a${}^{-1}$ in 2012 and continued losing mass because of global warming [57,58].
  A pair of S1A images covering the main part of Pine Island Glacier was download from the ESA Scientific Data Hub. Level-1 SLC data in interferometric wide swath mode were employed. The image pairs came from 36 -day repeat pass orbits (three repeat cycles), acquired on 17 February 2020 and 24 March 2020. The raw data were pre-processed using SARscape 5.2 software with geocoding of the intensity images to a 20 m × 20 m spatial resolution.

### 4.2. Experimental Details

In order to validate the reliability, the LUGC method and four other commonly used image correlation methods, namely NCC [28], OC [29], PC with the peak evaluation formula (PEF) [59], and PC with frequency optimization used in the COSI-Corr software (COSI-Corr-F) [60], were tested and compared in the applications of glacier surface motion estimation using SAR intensity images. OC, PEF, and COSI-Corr-F are image correlation methods operated in the frequency domain based on discrete FT, while NCC is directly calculated in the spatial domain by an iterative search process. The implementation of OC in ImGRAFT toolbox [61] and the implementation of NCC and COSI-Corr-F in the COSI-Corr software were adopted, and PEF was reimplemented by the authors.

The different image matching methods were assessed based on two criteria similar to [5]: matching accuracy and ability to obtain correct matches. To estimate the matching accuracy, two synthetic image pairs over glacierized regions were generated with ground truth displacements. The root mean squared error (RMSE) and mean absolute error between the derived displacements from different matching methods and ground truth displacements were calculated. The second criterion was calculated as the percentage of correct matches for each matching method. The assumed correct matches were identified by filtering the raw image matching results based on the prior motion, neighboring measurements, and connectivity analysis, as described in Section 3.1. The thresholds of the deviation between the raw matching result and the mean filtered matching result that were used to filter the 2D displacement maps in different test cases are listed in Table 2. This filtering operation is independent of the matching method used and has the same effect on different compared matching methods.

The template size plays a crucial role in correlation-based image matching. The selection of optimal template size should be large enough to ensure sufficient textural information for matching, but small enough to decrease the possibility of deformations in the template [62]. In this study, we empirically set the template size for each test case considering the image resolution and magnitude of displacements. The size of the matching windows and step of the matching grid in simulated and practical tests are also listed in Table 2. For Taku Glacier, the template sizes of both 32 pixels and 64 pixels were adopted to show the performance of different template sizes. For Pine Island Glacier, the images were matched in two steps that calculated the initial pixel-level movement using a large template (128 pixels), and then, the subpixel-level offset between the initially aligned templates with a small template size (64 pixels) was estimated. Note that the COSI-Corr-F method was initialized with a large template in both practical tests, since it cannot obtain satisfactory performance with small template sizes.

## 5. Results and Discussions

### 5.1. Experiments with Simulated Image Data

**Figure 4:**
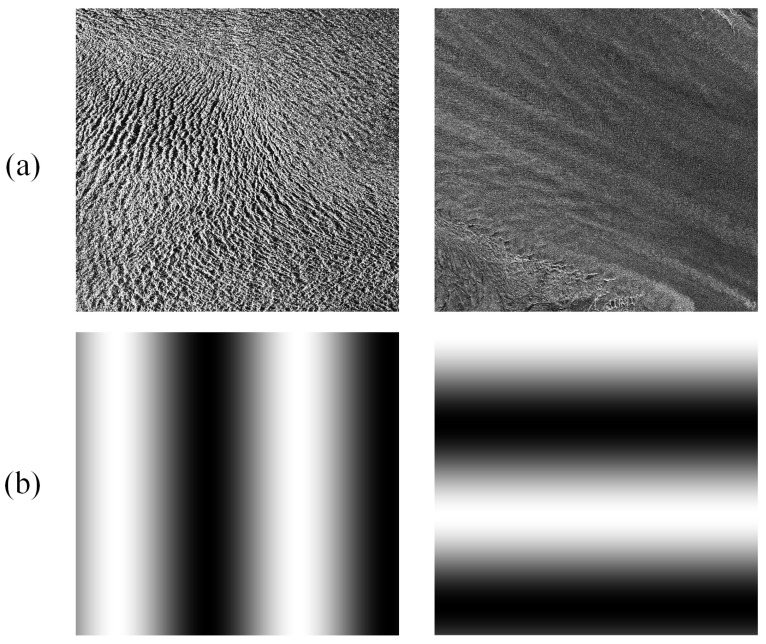
(**a**) TSX and S1A image subsets used to generate synthetic image pairs; (**b**) sinusoidal deformations in two directions.

The proposed method and other matching methods were firstly applied to the simulated SAR data to investigate the properties and matching accuracy in a controlled environment. Taking a subset of either TSX or S1A image over glacierized region as the master image of synthetic image pair (see Figure 4a), the slave image is constructed from the master image by adding a known displacement map in each direction through image resampling with a cubic interpolation. As shown in Figure 4b, different deformations both in the *x*- and *y*-direction with a sinusoidal transform were applied to model the nonlinear distortions. Here, the period of sinusoidal deformation was set as 900 pixels, and the amplitude was eight and six pixels in the *x*- and *y*-direction, respectively.

The results of different subpixel matching methods on these two synthetic image pairs in terms of mean absolute error and RMSE are given in Table 3. It can be seen that the matching errors are related to the amplitude of deformations as the *x*-direction errors of all the methods are higher than the *y*-direction ones. The rankings of matching methods in terms of mean absolute error and RMSE are the same. The LUGC method obtains the best matching results and the PEF method the worst results both when it comes to mean absolute error and RMSE. PC-based methods (i.e., PEF and COSI-Corr-F) generate more mismatches than the others, possibly due to the sensitivity of PC to local deformations in cases of a small template size [5]. On the contrary, the methods taking the image gradients into consideration (i.e., OC and LUCG) guarantee the promising robustness with the minimum number of mismatches. The results of the simulated SAR data demonstrate the effectiveness and superiority of the proposed LUGC method in subpixel capability and robustness to SAR intensity pattern and local deformation.

### 5.2. Experiments with Real Image Data

#### 5.2.1. Motion Estimation of Taku Glacier

**Figure 5:**
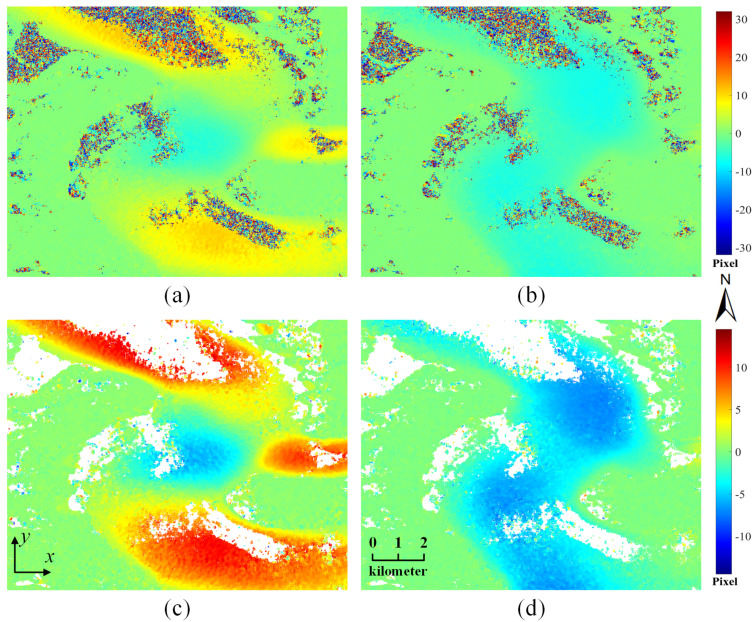
(**a**) *x*-direction and (**b**) *y*-direction displacement maps of the Taku Glacier derived from the LUGC method; (**c**) *x*-direction and (**d**) *y*-direction displacement maps after outlier removal.

After the pre-processing of TSX intensity images, the dense matching step was conducted between the orthorectified image pair to obtain the 2D displacements of the glacier surface. Figure 5a,b displays the *x*-direction and *y*-direction displacement maps of the Taku Glacier derived from the proposed LUGC method with a 64×64 template size. It is obvious that there exist mismatches in the matching results. These noisy estimates are mainly located in low-textured regions, such as snow-covered surfaces and shadowing on the glacier tongue, wherein adequate useful information to determine the correspondence between templates cannot be provided.

The post-processing step considering the displacement range, neighborhood, and connectivity was performed to filter out these erroneous measurements. Figure 5c,d shows the filtered results of *x*-direction and *y*-direction displacement maps in both glacierized and stable regions. As can be seen from the figures, most of the mismatches with abnormal values, inconsistent neighborhood, and isolated distribution are automatically eliminated by the post-processing step. The basic trends of the 2D surface motion for the Taku Glacier can be reflected after the outlier removal. The main trend of the glacier flow is from the northwest to the southeast direction, as the Taku Glacier is a tidewater glacier and the ice mass is flowing directly to the river. The surface displacements in the *y*-direction are relatively consistent downward, while the surface displacements in the *x*-direction show some local variations. Specifically, the flow direction in the middle part of the study area is reversed, probably because of the branch of Taku Glacier in the right part.

**Figure 6:**
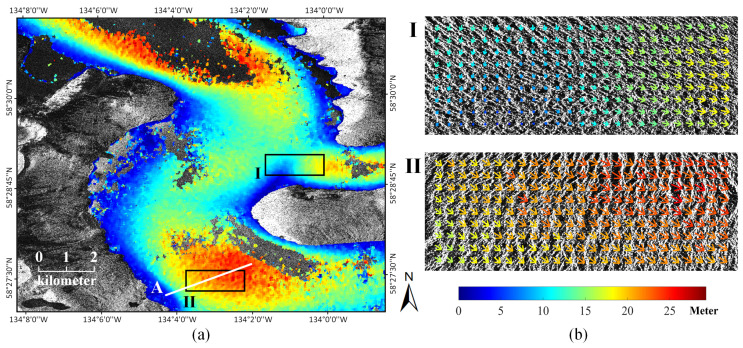
(**a**) Magnitudes of surface motions over the glacierized regions of the Taku Glacier; and (**b**) motion field of the glacier surface in the selected areas.

In Figure 6a, the magnitudes of surface motions over the glacierized regions were generated using the displacement maps and a manual labeled mask. It can be found that the maximal motion can reach approximately 25 m on the ground during the revisit period of 11 days. As shown in Figure 6b, motion fields of two representative areas were selected and illustrated with the direction of arrows indicating the direction of movement and the color indicating the magnitude of movement. In the first area, the motion field is locally anisotropic, and the turning of the flow direction can be observed. In the second area within the terminus region, the glacier moves in a coherent trend with the magnitude increasing from the boundary part to the central part.

#### 5.2.2. Motion Estimation of Pine Island Glacier

**Figure 7:**
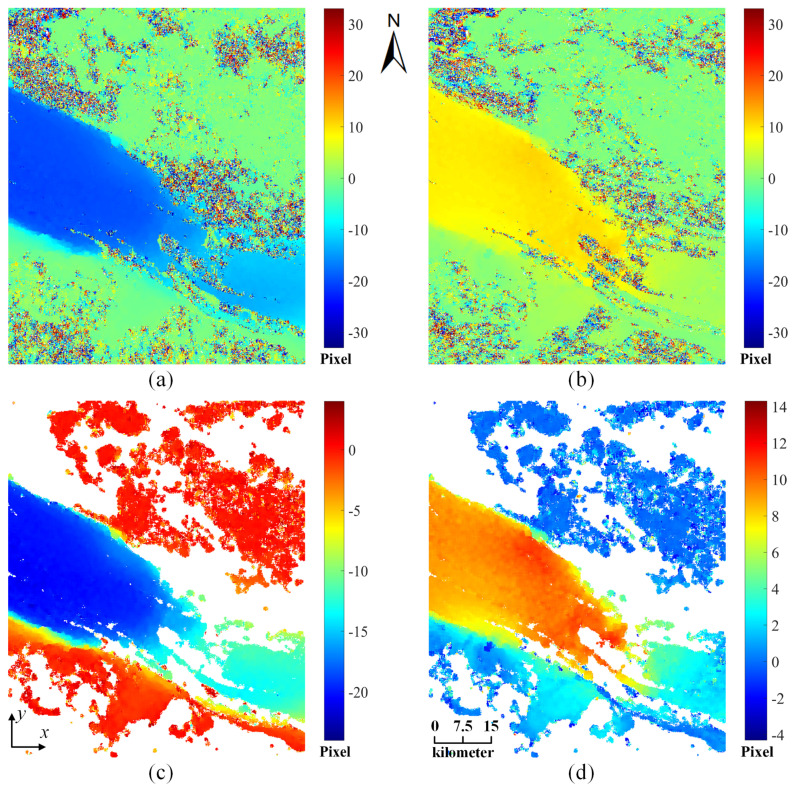
(**a**) x-direction and (**b**) y-direction displacement maps of the Pine Island Glacier derived from the LUGC method; (**c**) x-direction and (**d**) y-direction displacement maps after outlier removal.

Using the pre-processed multitemporal S1A intensity images, the *x*-direction and *y*-direction displacement maps of the Pine Island Glacier output by the dense matching step and the corresponding filtered displacement maps output by the post-processing step are presented in Figure 7a–d, respectively. It can be observed from Figure 7 that the automatic filtering operation successfully removes the erroneous measurements caused by poor visual contrast, mainly located in the parts surrounding Pine Island Glacier. The filtered displacement maps indicate that the ice streams in the central ice shelf flow from the southeast to the northwest direction and finally into Pine Island Bay, Amundsen Sea.

**Figure 8:**
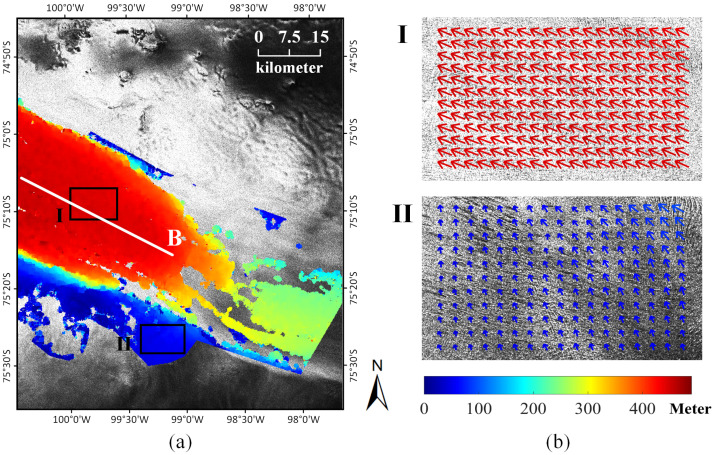
(**a**) Magnitudes of surface motions over the glacierized regions of the Pine Island Glacier; and (**b**) motion field of the glacier surface in the selected areas.

Similarly, the magnitudes of surface motions over the glacierized regions were composited using the displacement maps and a manual labeled mask, as shown in Figure 8a. As can be seen, the glacier flows mainly exist in the central ice shelf, and the motion in the front of the ice shelf is higher than other parts due to a rift and ice calving. To give a detailed view of glacier motion, the motion fields of two representative areas were also selected and illustrated in Figure 8b. The first area is located on the central ice shelf with consistently high ice velocities. The maximal motion can reach around 450 m on the ground, and the ice velocity equals 375 m/month considering the interval of 36 days, which is similar to the value in [63] acquired by the InSAR technique. The second area is located on the southern ice shelf wherein a slow-moving tributary exists flowing into the central ice shelf with a maximal velocity of around 65 m/month. Both motion fields in the central ice shelf and the southern ice shelf show a coherent trend.

#### 5.2.3. Comparison with Other Matching Methods

The displacement maps of the Taku Glacier and Pine Island Glacier were calculated and filtered using different matching methods and the same pre-processing and post-processing workflow. The numbers of assumed correct matches that passed the filtering operation both over the glacierized regions and over the entire image were counted. The percentages of correct matches for different matching methods are given in Table 4.

It is obvious that the results vary both from region-to-region and from method-to-method. The percentages of correct matches in Taku Glacier are higher than ones in Pine Island Glacier for all matching methods. This is because that Pine Island Glacier has more snow-covered areas especially in the surrounding regions. The textureless areas easily generate noisy matching measurements, which are removed by the filtering operation.

Among the compared matching methods, the proposed LUGC method generally outperforms other matching methods and obtains the best performance of the assumed corrected matches after the same filtering operation in all cases regardless of the test regions and template sizes. The higher percentages of correct matches indicate that the LUGC method is able to provide stable and consistent results of glacier motion estimation. The robust performance is attributed to the joint use of the magnitude and orientation information of edge responses and frequency-based local upsampling, which ensure image correlation with salient structural frequency components and efficient subpixel calculation. In contrast, the PC-based methods perform the worst. The PEF method obtains the lowest percentages of correct matches, and the COSI-Corr-F method can only generate reasonable results for Taku Glacier with an initialization using a larger template. This agrees with the situation in the simulated test, but is opposed to the situation in [5], which found that the COSI-Corr-F method can achieve the best and most robust performance when using optical Landsat data for glacier motion estimation. This implies that PC is susceptible to SAR intensity noise and is unsuitable for matching SAR intensity images.

In addition, it is worth noting that the results for Taku Glacier derived using template sizes of 64 pixels are largely improved compared to those using template sizes of 32 pixels. This demonstrates the significance and difficulty of selecting optimal template sizes. However, the requirements of template size to obtain valid results are diverse for different grid positions, since a small template size is adequate for certain positions. Therefore, an adaptive strategy for adjusting template size based on local structural statistics is highlighted [62,64]. Future work will explore the combination of the presented LUGC method with adaptive template strategies to ensure both the correctness in low textured areas and the locality in areas with strong velocity gradient.

**Figure 9:**
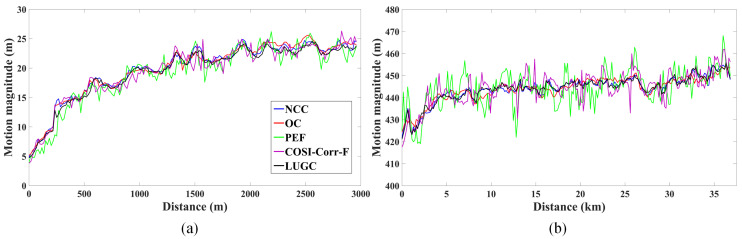
Magnitudes of surface motions along the two marked profiles. (**a**) Profile A; and (**b**) Profile B.

To show the performance of different methods in the key part of the glacier surface, each profile was selected in Taku Glacier and Pine Island Glacier as shown in Figure 6a and Figure 8a. Profile A is located at the terminus region of Taku Glacier with a length of around 3 km, and Profile B is located at the central ice shelf of Pine Island Glacier with a length of around 37 km. Figure 9 displays the magnitudes of surface motions along the profiles derived from different matching methods. A simple visual inspection of the figures shows that all the matching methods can capture the similar underlying tendency of glacier surface motion. For Profile A, the plots of motion magnitude reveal an increasing trend from below 5 m in the boundary part to around 25 m in the central part. For Profile B, the plots of motion magnitude present a stable varying tend in the range between 420 m and 460 m. Moreover, the comparison indicates that the results of the NCC and LUGC methods are relatively smoother with less frequent fluctuations and unexpected spikes caused by the matching errors.

## 6. Conclusions

This paper presents a motion estimation method of glacier surface using repeated SAR intensity images based on subpixel gradient correlation. The overall workflow of glacier motion estimation includes pre-processing, dense matching, and post-processing steps. A robust frequency-based image correlation method is developed by locally upsampling the correlation function calculated from the complex representations of image gradients in the frequency domain, which can effectively achieve subpixel dense matching of SAR intensity images. The proposed method is validated using simulated and real SAR data. The monitoring of Taku Glacier and Pine Island Glacier between revisit periods of SAR data is successfully conducted with the motion obtained and the trend investigated. The comparison with commonly used correlation-based matching methods demonstrates that the proposed LUGC method is able to achieve higher matching accuracy, as well as generate higher percentages of correct matches and more stable and smoother motion results. In future work, the presented method will be extended to more detailed applications with multi-pass SAR intensity data, and the improvement of the matching method will be investigated by combining adaptive template strategies and considering better robustness against SAR speckle noises.

## Figures and Tables

**Table 1:** TerraSAR-X (TSX) and Sentinel-1A (S1A) image data used in the experiments.

Image Data	Acquisition Date	Resampled Resolution	Imaging Mode	Data Type	Polarization	DEM Used
TSX	11 July 2009 22 July 2009	2.09 m × 2.09 m	Strip-map	SLC	HH	SRTM DEM 30 m × 30 m
S1A	17 February 2020 24 March 2020	20 m × 20 m	Interferometric wide swath	SLC	HH	ICESAT DEM 500 m × 500 m

**Table 2:** Template size, grid step, and filtering threshold in different test cases (unit: pixels).

Test	Template Size	Step	Filtering Threshold
Simulated	32	2	0.5
Take	32/64	8	3
Pine Island	128-64	8	4

**Table 3:** Mean absolute error and RMSE of the matching results of different matching methods. NCC (normalized cross-correlation); OC (orientation correlation); PEF (peak evaluation formula); COSI-Corr-F (phase correlation in COSI-Corr).

Method	Mean Absolute Error (pixels)		RMSE (pixels)		Number of Mismatches
	*x*-direction	*y*-direction	*x*-direction	*y*-direction	
NCC	0.149	0.097	0.180	0.123	17
OC	0.143	0.091	0.164	0.109	8
PEF	0.162	0.139	0.197	0.175	58
COSI-Corr-F	0.132	0.084	0.155	0.103	99
LUGC	0.090	0.063	0.112	0.080	8

**Table 4:** RMSE and mean absolute error of the matching results of different matching methods.

Method	Taku Glacier				Pine Island Glacier	
	32		64		128-64	
	Glacier	All	Glacier	All	Glacier	All
NCC	56%	58.9%	72.7%	76.3%	65.9%	45.4%
OC	53.7%	54.6%	69.4%	72.9%	62.4%	40.3%
PEF	41.1%	48.3%	60.7%	68.6%	54.4%	35.9%
COSI-Corr-F	59.9%	65.4%	63.2%	69.8%	58.1%	38.7%
LUGC	58.1%	63.9%	75.8%	80.4%	70%	51.8%
